# Projecting the Impact of SARS-CoV-2 Variants and the Vaccination Program on the Fourth Wave of the COVID-19 Pandemic in South Korea

**DOI:** 10.3390/ijerph18147578

**Published:** 2021-07-16

**Authors:** Eunha Shim

**Affiliations:** Department of Mathematics, Soongsil University, Seoul 06978, Korea; alicia@ssu.ac.kr; Tel.: +82-(02)-820-0146

**Keywords:** COVID-19, SARS-CoV-2, vaccine, Korea, vaccine allocation strategy, herd immunity, mathematical model

## Abstract

Vaccines against severe acute respiratory syndrome coronavirus 2 (SARS-CoV-2) are currently administered in South Korea; however, vaccine supply is limited. Considering constraints in vaccine supply and the emergence of variant strains, we evaluated the impact of coronavirus disease (COVID-19) vaccination program in reducing incidence, ICU hospitalization, and deaths in South Korea. We developed an age-structured model of SARS-CoV-2 transmission parameterized with Korean demographics and age-specific COVID-19 outcomes. Using our model, we analyzed the impact of the COVID-19 vaccination program during the fourth wave of the pandemic in South Korea in reducing disease burden. We projected that the vaccination program can reduce the overall attack rate to 3.9% from 6.9% without vaccination, over 150 days, starting from 5 July 2021. The highest relative reduction (50%) was observed among individuals aged 50–59 years. Vaccination markedly reduced adverse outcomes, such as ICU hospitalizations and deaths, decreasing them by 45% and 43%, respectively. In the presence of the Delta variant, vaccination is expected to reduce the overall attack rate to 11.9% from 26.9%. Our results indicate that the impact of vaccination can be substantially affected by the emergence of SARS-CoV-2 variants. Furthermore, herd immunity is unlikely to be achieved with the potential emergence of the Delta variant, inconsistent with the blueprint of the South Korean government.

## 1. Introduction

Despite unprecedented social distancing measures and vaccine development efforts, the rapid spread of coronavirus disease (COVID-19) continues to impose a heavy burden on healthcare at a global scale, resulting in 183 million reported cases and 3.9 million deaths worldwide, as of 4 July 2021 [1]. Since the first case of severe acute respiratory syndrome coronavirus 2 (SARS-CoV-2) was reported in January 2020, the virus has been constantly evolving, and several variants have emerged with mutations that alter the receptor binding domain of the spike protein [1,2]. Certain variants, called variants of concern (VOC), have shown their rapid emergence within populations, with increased potential for transmission or clinical implications, including the Alpha variant first identified in the United Kingdom (UK), the Beta variant in South Africa, the Gamma variant in Brazil, and the Delta variant in India [3]. Among these variants, the SARS-CoV-2 variant Alpha, also known as B.1.1.7, is estimated to have emerged in the UK in September 2020 and has become the dominant circulating SARS-CoV-2 variant in England [3]. The Alpha variant is found to have a selective advantage over the other variants, being 56% more transmissible (95% confidence interval (CI): 50–74%) across three regions, i.e., Denmark, Switzerland, and the United States (US), compared to the pre-existing variants of SARS-CoV-2 [4,5,6]. Furthermore, the Delta variant, also known as B.1.617.2, was first identified in India in December 2020 and has now spread to South Korea. Data from the UK suggest that the Delta variant is 40–60% more transmissible than the Alpha variant.

In South Korea, the detection rate of major virus variants (Alpha, Beta, Gamma, and Delta) in community transmission in June 2021 was over 30%; meanwhile, the cases of Delta variant have surged [7]. Furthermore, on 7 July 2021, it was announced that the fourth wave has hit South Korea, with more than 1200 new daily cases reported on average in July. The Korea Disease Control and Prevention Agency (KDCA) announced that over the first week of July 2021, 26.5% of newly confirmed COVID-19 patients in the capital area were infected with the Delta variant, expressing concern that the Delta variant cases may become prevalent in the country [7].

All authorized COVID-19 vaccines have demonstrated an efficacy of 65–95% against symptomatic, laboratory-confirmed COVID-19 caused by the non-variant strain [2]. However, the efficacy of COVID-19 vaccines might be potentially affected by the emergence of mutant SARS-CoV-2 variants, which are potentially more virulent and have increased infectivity. It has been suggested that the currently authorized COVID-19 vaccines may provide some protection against the various strains, including the Alpha variant [8]. However, reduced antibody neutralization and efficacy have been observed for the Delta variant.

Specifically, there is no evidence that the efficacy of Moderna or Pfizer COVID-19 vaccines is reduced against the Alpha variant [8]. In the UK, when the Alpha variant was the predominant circulating strain, two doses of the Pfizer COVID-19 vaccine demonstrated an efficacy of 85–86% against SARS-CoV-2 infection and symptomatic COVID-19 [2]. Novavax announced that its vaccine showed 85% efficacy against the Alpha variant versus 89% with the non-variant strain, while the AstraZeneca-Oxford vaccine reportedly offered 74% efficacy against the Alpha variant and 84% against the non-variant strain [6]. However, the efficacy of vaccines against the Delta variant is potentially reduced. The clinical trials of the Pfizer and AstraZeneca-Oxford COVID-19 vaccines showed reduced sera neutralization for the Delta variant, with a protective efficacy of 79% and 60% against this variant, respectively [8,9]. However, the protective efficacy of the Pfizer and AstraZeneca-Oxford vaccine was lower with a single dose, with 36% and 30% efficacy against symptomatic diseases by the Delta variant, respectively [10].

As SARS-CoV-2 variants continue to spread globally, it is imperative to assess their impact on vaccination programs at the population level. In this study, we constructed an age-structured, two-strain model of SARS-CoV-2 transmission and vaccination, and applied our model to the South Korean population, where domestic transmission of the Delta variant has been increasingly reported. We examined the impact of a novel variant on vaccination campaigns at the population level during the fourth wave of the pandemic, under various scenarios related to the transmission potential and the emergence of novel variants. Based on our simulation results, we examined whether herd immunity could be achieved with current vaccination programs in the presence of the Delta variant.

## 2. Materials and Methods

### 2.1. Model Structure

**Figure 1:**
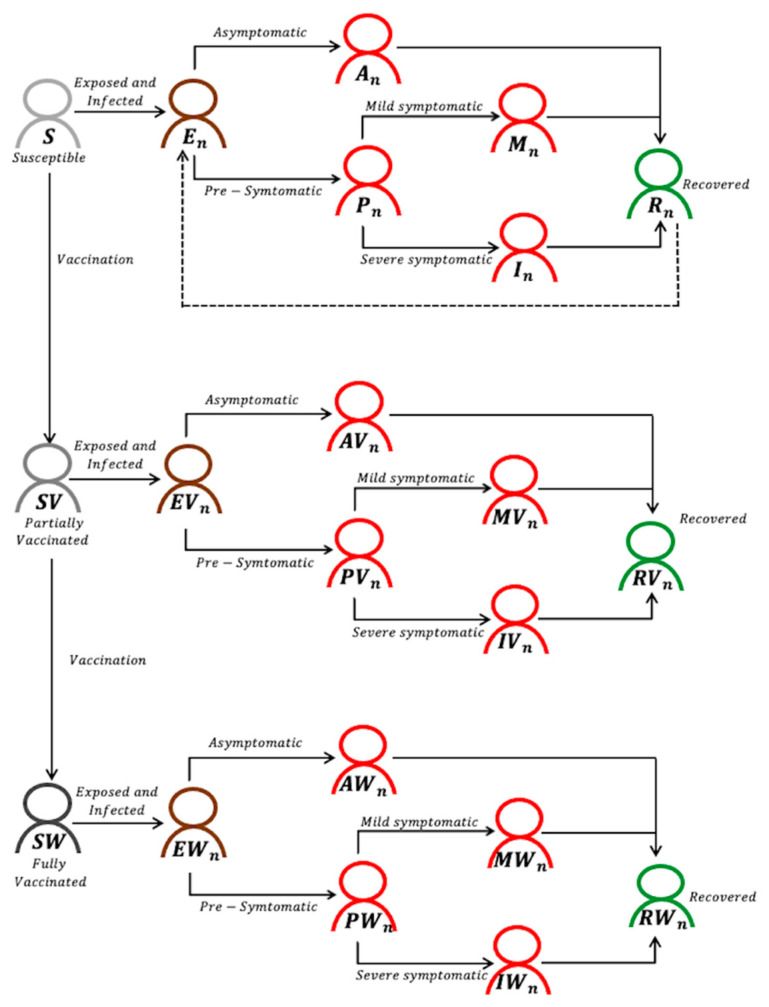
Model diagram of COVID-19 transmission and vaccination with two co-circulating strains, denoted by *n* (*n* = 1 or 2). All individuals are stratified by age, although age indices have been omitted for clarity.

We developed an age-structured model of COVID-19 transmission and vaccination considering two co-circulating strains (Figure 1). Our model assumed that the mutant strain would be more transmissible on contact with an infectious person than the pre-existing, wild-type strain, i.e., υ>1. The model classified individuals according to their vaccination status and the natural history of COVID-19, considering the two strains. Additionally, as unvaccinated individuals are susceptible to both the wild and mutant strains, they were classified as follows: susceptible ($S_{k}$); exposed ($E_{j,k}$), asymptomatic ($A_{j,k}$); pre-symptomatic ($P_{j,k}$); mildly symptomatic ($M_{j,k}$); severely symptomatic ($I_{j,k}$); and recovered ($R_{j,k}$). Similarly, partially (or fully) vaccinated individuals are divided into susceptible ($SV_{k}\;$ or $SW_{k}$), exposed ($EV_{j,k}$ or $EW_{j,k}$), asymptomatic ($AV_{j,k}$ or $AW_{j,k}$), pre-symptomatic ($PV_{j,k}$ or $PW_{j,k}$), mildly symptomatic ($MV_{j,k}$ or $MW_{j,k}$), severely symptomatic ($IV_{j,k}$ or $IW_{j,k}$), and recovered ($RV_{j,k}$ or $RW_{j,k}$) categories. Here, the subscript *k* refers to the age group of individuals (*k* = 1, 2, …, 8), while the subscript *j* indicates the original strain (*j* = 1) or variant alpha (*j* = 2). Model population was stratified into eight age groups of 0–9, 10–19, 20–29, 30–39, 40–49, 50–59, 60–69, and 70+ years based on the Korean census data.

Vaccinated individuals followed the analogous natural history of COVID-19, although they had a lower probability of both clinical and subclinical infection. However, it was assumed that vaccinated individuals who develop clinical or subclinical infection would be as infectious as unvaccinated individuals with clinical or subclinical infection. We parameterized our model with daily contacts within and between age groups using synthetic country-specific contact matrices that were constructed based on the POLYMOD study [11,12]. Our simulations were run with and without the introduction of SARS-CoV-2 variants. For cases with SARS-CoV-2 variants, we assumed that Delta would be introduced on day 25 (i.e., 30 July 2021), when school holidays and summer vacation would begin in South Korea. Our mathematical model with two strains of SARS-CoV-2 and vaccination is given by the following equations:

$$\begin{array}{c} S_{k}{}^{\prime }=-\left(\theta_{k}\left(t\right)+\lambda_{1,k}\left(t\right)+\lambda_{2,k}\left(t\right)\right)\;S_{k},\\ E_{1,k}{}^{\prime }=\lambda_{1,k}\left(t\right)S_{k}-\gamma_{E}E_{1,k},\\ A_{1,k}{}^{\prime }=\left(1-h_{k}\right)\gamma_{E}E_{1,k}-\gamma_{A}A_{1,k},\\ P_{1,k}{}^{\prime }=h_{k}\gamma_{E}E_{1,k}-\gamma_{P}P_{1,k},\\ M_{1,k}{}^{\prime }=\rho_{k}\gamma_{P}P_{1,k}-\gamma_{M}M_{1,k},\\ I_{1,k}{}^{\prime }=\left(1-\rho_{k}\right)\gamma_{P}P_{1,k}-\gamma_{I}I_{1,k},\\ R_{1,k}{}^{\prime }=\gamma_{A}A_{1,k}+\gamma_{M}M_{1,k}+\gamma_{I}I_{1,k}-\sigma \lambda_{2,k}\left(t\right)R_{1},\\ E_{2,k}{}^{\prime }=\lambda_{2,k}S_{k}+\sigma \lambda_{2,k}\left(t\right)R_{1}-\gamma_{E}E_{2,k},\\ A_{2,k}{}^{\prime }=\left(1-h_{k}\right)\gamma_{E}E_{2,k}-\gamma_{A}A_{2,k},\\ P_{2,k}{}^{\prime }=h_{k}\gamma_{E}E_{2,k}-\gamma_{P}P_{2,k},\\ M_{2,k}{}^{\prime }=\rho_{k}\gamma_{P}P_{2,k}-\gamma_{M}M_{2,k},\\ I_{2,k}{}^{\prime }=\left(1-\rho_{k}\right)\gamma_{P}P_{2,k}-\gamma_{I}I_{2,k},\\ R_{2,k}{}^{\prime }=\gamma_{A}A_{2,k}+\gamma_{M}M_{2,k}+\gamma_{I}I_{2,k},\\ SV_{k}{}^{\prime }=\theta_{k}\left(t\right)S_{k}-\left\{\left(1-\eta_{1,k}\right)\lambda_{1,k}\left(t\right)+\left(1-\eta_{2,k}\right)\lambda_{2,k}\left(t\right)\right\}SV_{k}-\psi_{k}\left(t\right)\;SV_{k},\\ EV_{1,k}{}^{\prime }=\left(1-\eta_{1,k}\right)\lambda_{1,k}SV_{k}-\left\{\left(1-h_{k}\right)+\left(1-\delta_{1,k}\right)h_{k}\right\}\gamma_{E}EV_{1,k},\\ AV_{1,k}{}^{\prime }=\left(1-h_{k}\right)\gamma_{E}EV_{1,k}-\gamma_{A}AV_{1,k},\\ PV_{1,k}{}^{\prime }=\left(1-\delta_{1,k}\right)h_{k}\gamma_{E}EV_{1,K}-\gamma_{P}PV_{1,K},\\ MV_{1,k}{}^{\prime }=\rho_{k}\gamma_{P}PV_{1,k}-\gamma_{M}MV_{1,k},\\ IV_{1,k}{}^{\prime }=\left(1-\rho_{k}\right)\gamma_{P}PV_{1,k}-\gamma_{I}IV_{1,k},\\ RV_{1,k}{}^{\prime }=\gamma_{A}AV_{1,k}+\gamma_{M}MV_{1,k}+\gamma_{I}IV_{1,k},\\ EV_{2,k}{}^{\prime }=\left(1-\eta_{2,k}\right)\lambda_{2,k}\left(t\right)SV_{k}-\left\{\left(1-h_{k}\right)+\left(1-\delta_{1,k}\right)h_{k}\right\}\gamma_{E}EV_{2,k},\\ AV_{2,k}{}^{\prime }=\left(1-h_{k}\right)\gamma_{E}EV_{2,k}-\gamma_{A}AV_{2,k},\\ PV_{2,k}{}^{\prime }=\left(1-\delta_{2,k}\right)h_{k}\gamma_{E}EV_{2,k}-\gamma_{P}PV_{2,k},\\ MV_{2,k}{}^{\prime }=\rho_{k}\gamma_{P}PV_{2,k}-\gamma_{M}MV_{2,k},\\ IV_{2,k}{}^{\prime }=\left(1-\rho_{k}\right)\gamma_{P}PV_{2,k}-\gamma_{I}IV_{2,k},\\ RV_{2,k}{}^{\prime }=\gamma_{A}AV_{2,k}+\gamma_{I}IV_{2,k}\\ SW_{k}{}^{\prime }=\psi_{k}\left(t\right)\;SV_{k}-\left\{\left(1-\alpha_{1,k}\right)\lambda_{1,k}\left(t\right)+\left(1-\alpha_{2,k}\right)\lambda_{2,k}\left(t\right)\right\}SW_{k},\\ EW_{1,k}{}^{\prime }=\left(1-\alpha_{1,k}\right)\lambda_{1,k}SW_{k}-\left\{\left(1-h_{k}\right)+\left(1-\chi_{1,k}\right)h_{k}\right\}\gamma_{E}EW_{1,k},\\ AW_{1,k}{}^{\prime }=\left(1-h_{k}\right)\gamma_{E}EW_{1,k}-\gamma_{A}AW_{1,k},\\ PW_{1,k}{}^{\prime }=\left(1-\chi_{1,k}\right)h_{k}\gamma_{E}EW_{1,K}-\gamma_{P}PW_{1,K},\\ MW_{1,k}{}^{\prime }=\rho_{k}\gamma_{P}PW_{1,k}-\gamma_{M}MW_{1,k},\\ IW_{1,k}{}^{\prime }=\left(1-\rho_{k}\right)\gamma_{P}PW_{1,k}-\gamma_{I}IW_{1,k},\\ RW_{1,k}{}^{\prime }=\gamma_{A}AW_{1,k}++\gamma_{M}MW_{1,k}+\gamma_{I}IW_{1,k},\\ EW_{2,k}{}^{\prime }=\left(1-\alpha_{2}\right)\lambda_{2,k}SW_{k}-\left(1-\chi_{2,k}\right)\gamma_{E}EW_{2,k},\\ AW_{2,k}{}^{\prime }=\left(1-h_{k}\right)\gamma_{E}EW_{2,k}-\gamma_{A}AW_{2,k},\\ PW_{2,k}{}^{\prime }=\left(1-\chi_{2,k}\right)h_{k}\gamma_{E}EW_{2,K}-\gamma_{P}PW_{2,K},\\ MW_{2,k}{}^{\prime }=\rho_{k}\gamma_{P}PW_{2,k}-\gamma_{M}MW_{2,k},\\ IW_{2,k}{}^{\prime }=\left(1-\rho_{k}\right)\gamma_{P}PW_{2,k}-\gamma_{I}IW_{2,k},\\ RW_{2,k}{}^{\prime }=\gamma_{A}AW_{2,k}++\gamma_{M}MW_{2,k}+\gamma_{I}IW_{2,k}, \end{array}$$

where

$$\lambda_{1,k}=\beta_{k}\sum_{j=1}^{16}c_{kj}\left(\frac{q_{A}\left(A_{1,j}+AV_{1,j}+AW_{1,j}\right)+(P_{1,j}+PV_{1,j}+PW_{1,j})+q_{M}\left(M_{1,j}+MV_{1,j}+MW_{1,j}\right)+q_{I}\left(I_{1,j}+IV_{1,j}+IW_{1,j}\right)}{N_{j}}\right)\;\mathrm{and}\;\lambda_{2,k}=\upsilon \lambda_{1,k}.$$

### 2.2. Disease Dynamics

In our mathematical model, the risk of infection for people susceptible to COVID-19 depended on contact with individuals infected by pre-existing wild-type or mutant SARS-CoV-2 that could be in asymptomatic, pre-symptomatic, or symptomatic stages of the disease. Our model assumes that individuals with clinical symptoms are more infectious than asymptomatic individuals [13]. Specifically, it was assumed that the infectivity of asymptomatic, mild symptomatic, and severely symptomatic individuals would be 26%, 22% and 44%, respectively, relative to the pre-symptomatic stage, considering the impact of self-isolation of symptomatic patients (Table 1) [14,15,16,17]. For infected individuals, the average incubation period was assumed to be 5.2 days, and an age-specific proportion ($h_{k}$) of infected individuals were assumed to develop symptomatic disease following a pre-symptomatic stage [18,19]. The pre-symptomatic stage and infectious period were assumed to be 2.3 days and 3.2 days on average, respectively [18,19]. Those who did not develop symptomatic disease were assumed to remain asymptomatic until recovery, with an average infectious period of 5 days [20,21]. Symptomatic cases would have an age-specific probability of developing mild or severe illness. We assumed that individuals who recovered from infection by pre-existing original strain of SARS-CoV-2 were partially susceptible to infection by SARS-CoV-2 variant.

### 2.3. Vaccination

As of 5 July 2021, two COVID-19 vaccines (i.e., AstraZeneca-Oxford and Pfizer COVID-19 vaccines) have been widely used in South Korea: a viral vector vaccine with lower efficacy against symptomatic disease and an mRNA vaccine that demonstrated high efficacy against symptomatic disease [22,23,24]. Specifically, as of 5 July 2021, 19,537,712 doses of approved COVID-19 vaccines have been administered in South Korea, including 11,366,200 (58.2%) doses of the AstraZeneca-Oxford vaccine and 7,002,955 (35.8%) doses of the Pfizer vaccine [25].

Our simulation approach is modeled after the vaccination program in South Korea (Table 2). Vaccines were modeled as a two-dose regimen with 19.5% and 10% of the population being partially and fully vaccinated, respectively, on day 1 (5 July 2021) [25]. In baseline vaccination scenarios, vaccination was assumed to continue according to the current rollout plan, and we examined its potential impact on symptomatic disease, hospitalizations, and deaths. Vaccine administration in South Korea is partly constrained due to the limited vaccine supply and the capacity to administer doses. In addition, vaccination against COVID-19 is performed in order of decreasing age, with the minimum age being 18. In our model, vaccines were administered to all adults aged ≥20 years in descending order of age group. As of 5 July 2021, 84% of individuals aged ≥60 years received at least one dose of COVID-19 vaccine. Thus, in our model, unvaccinated individuals aged ≥60 years were vaccinated with the first dose until the coverage level of 87% was reached, following which vaccines were administered to individuals aged 40–59 years and then to those aged 20–39 years starting on August 1 and September 1, respectively. All susceptible individuals were assumed to be eligible for vaccination. The detailed supply schedule used in this analysis was based on public announcements [26]. Specifically, the constant daily rate of vaccine administration in younger (aged 20–39) and older adults (aged 40–59) was scaled to meet the target vaccine coverage levels of 86% and 76% by day 90 (25 September), respectively. With this rollout plan, we predicted to achieve 70% coverage level of the entire population by 25 September 2021. This rate corresponds to the goal of vaccination of 36 million individuals with the first dose by the end of September 2021, as outlined by the KDCA [26]. In addition, we assumed that the vaccination campaign with the second dose would start on day 95, 110, and 120 for individuals aged ≥60, 40–59 years, and 20–39 years, respectively. The constant daily rate of vaccine administration was scaled to achieve 65% of second-dose coverage level of the entire population by 30 November 2021. Infection dynamics continued during the simulation, and outcomes were evaluated for 150 days.

We assumed “leaky” vaccine protection in which all vaccinated individuals were partially protected against infection and subject to some residual risk of infection and symptomatic disease. This contrasts with an “all-or-none” vaccine efficacy, in which a proportion of vaccinated individuals would have complete protection. The baseline values of vaccine efficacy used in the model are listed in Table 1. The overall vaccine effectiveness against disease (denoted by *VE*) was modeled as a function of the risk of infection and risk of symptomatic disease, conditional to infection [27]. Specifically, the vaccine effectiveness ($VE_{j,k}$) against strain *j* (*j* = 1 for wild-type and *j* = 2 for SARS-CoV-2 variant) among age group *k* is represented as

$$VE_{j,k}=1-\left(1-\eta_{j,k}\right)\left(1-\delta_{j,k}\right)$$

where $\eta_{j,k}$ represents vaccine efficacy against infection and $\delta_{j,k}$ represents the efficacy against symptomatic disease, conditional to infection. First-dose and second-dose effectiveness values (Table 1) were based on estimates from prior studies where extended dose interval strategy was employed [28,29]. We assumed that vaccine effectiveness after the first and the second dose in preventing symptomatic disease due to infection with the wild type, $VE_{1,k}$ (*k* = 3, …, 6), was 70% and 80%, respectively, reflecting the efficacy of the AstraZeneca or Pfizer vaccine [22,23,24,28,29,30]. An additional scenario with the emergence of the Delta variant was evaluated, considering the efficacy after receiving one dose and two doses of the AstraZeneca or Pfizer vaccine (Table 1) [22,24]. Based on a prior study, we assumed that the vaccine efficacy against the Delta variant, $VE_{2,k}$ (*k* = 3, …, 6), after receiving a single dose and two doses of the AstraZeneca or Pfizer vaccine is 32% and 65%, respectively (Table 1). For all scenarios, vaccine efficacy was assumed to be reduced by a factor of 30% in vaccinated individuals aged ≥60 years, based on observed reduction in vaccine effectiveness among older individuals [31]. In the absence of data for vaccine efficacy against infection or transmission, we assumed that vaccination provides protection against symptoms of both the pre-existing original strain and the Delta variant with 50% lower efficacy than its efficacy against infection (Table 1). We assumed that the vaccine efficacy would not wane during the relatively short time span used for this analysis (150 days).

**Table 1:** Description of the model parameters and their estimates.

Notation	Description	Estimates	References
$1/\gamma_{E}$	Incubation period (days)	5.2 days	[18]
$1/\gamma_{A}$	Asymptomatic period (days)	5.0 days	[16,20,21]
$1/\gamma_{P}$	Pre-symptomatic period (days)	2.3 days	[14,16,19]
$1/\gamma_{M}$	Infectious period from onset of mild symptoms (days)	3.2 days	[16,20]
$1/\gamma_{S}$	Infectious period from onset of severe symptoms (days)	3.2 days	[16,20]
1−σ	Reduction of infection by variant strain due to cross immunity	0.7	[32]
υ	Relative transmission potential of strain 2 compared to strain 1	2	[10]
$q_{A}$	Relative infectivity of asymptomatic individuals compared to the pre-symptomatic stage	0.26	[15]
$q_{M}$	Relative infectivity of mildly symptomatic individuals compared to the pre-symptomatic stage, considering isolation	0.22	[14,17,33]
$q_{I}$	Relative infectivity of severely symptomatic individuals compared to the pre-symptomatic stage, considering isolation	0.44	[14,17,33]
$h_{k}$	Proportion of infections that are symptomatic	0.29 for *k* = 1; 0.21 for *k* = 2; 0.27 for *k* = 3; 0.33 for *k* = 4; 0.40 for *k* = 5; 0.49 for *k* = 6; 0.63 for *k* = 7; 0.69 for *k* = 8	[13]
$\rho_{k}$	Proportion of symptomatic cases that exhibit mild symptoms	0.90 for *k* = 1 and 2; 0.85 for *k* = 3, 4, and 5; 0.60 for *k* = 6 and 7; 0.20 for *k* = 8	[34,35]
$\eta_{1,k}$	Vaccine efficacy, after the first dose to before the second dose, against infection by strain 1 among the age group *k*	0.53 for *k* = 1, 2, …, 6; 0.37 for *k* = 7 and 8	[22,23,24,31]
$\eta_{2,k}$	Vaccine efficacy, after the first dose to before the second dose, against infection by strain 2 among the age group *k*	0.21 for *k* = 1, 2, …, 6; 0.14 for *k* = 7 and 8	[10,29,36,37]
$\delta_{1,k}$	Vaccine efficacy, after the first dose to before the second dose, against symptomatic disease by strain 1 among the age group *k*	0.58 for *k* = 1, 2, …, 6; 0.41 for *k* = 7 and 8	[22,23,24,31]
$\delta_{2,k}$	Vaccine efficacy, after the first dose to before the second dose, against symptomatic disease by strain 2 among the age group *k*	0.14 for *k* = 1, 2, …, 6; 0.10 for *k* = 7 and 8	[10,29,36,37]
$\alpha_{1,k}$	Vaccine efficacy, after the second dose, against infection by strain 1 among the age group *k*	0.64 for *k* = 1, 2, …, 6; 0.37 for *k* = 7 and 8	[38,39]
$\alpha_{2,k}$	Vaccine efficacy, after the second dose, against infection by strain 2 among the age group *k*	0.48 for *k* = 1, 2, …, 6; 0.33 for *k* = 7 and 8	[8,9]
$\chi_{1,k}$	Vaccine efficacy, after the second dose, against symptomatic disease by strain 1 among the age group *k*	0.37 for *k* = 1, 2, …, 6; 0.26 for *k* = 7 and 8	[22,23,24,31]
$\chi_{2,k}$	Vaccine efficacy, after the second dose, against symptomatic disease by strain 2 among the age group *k*	0.33 for *k* = 1, 2, …, 6; 0.23 for *k* = 7 and 8	[8,9]

### 2.4. Model Implementation

For model calibration, an effective reproduction number of 1.2 was used to account for the effect of current non-pharmaceutical COVID-19 interventions in South Korea [25]. To incorporate the age distribution of pre-existing immunity in the population, the information on case distribution as well as vaccinated proportions in age groups were used as the initial conditions (Table 2). We simulated the model across a time span of 150 days, assuming pre-existing natural and vaccine-induced immunity at the start of vaccination.

**Table 2:** Cumulative percentage of people who have received a COVID-19 vaccine in South Korea by vaccination status and age group as of 5 July 2021 [25].

		Partially Vaccinated (%)	Fully Vaccinated (%)
Total		19.5	10.4
Age group	20–29	6.4	4.1
	30–39	16.4	14.4
	40–49	9.0	4.7
	50–59	8.6	3.6
	60–69	79.9	3.3
	70 and above	33.6	51.1

## 3. Results

The transmission probability per contact was calibrated to an effective reproduction number *R*_e_ = 1.2. For the base-case scenario without the emergence of a SARS-CoV-2 variant, the attack rate was projected to be 6.9% on day 150 without further vaccination.

### 3.1. Attack Rates

**Figure 2:**
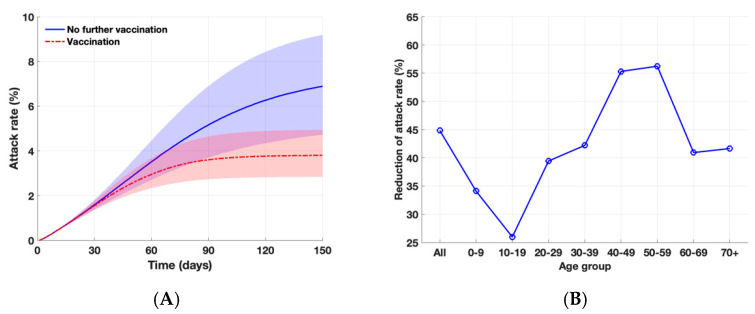
Impact of vaccination program against COVID-19 during the fourth wave in the absence of SARS-CoV-2 variant. (**A**) Projected cumulative attack rates over 150 days with and without further vaccination. The shaded areas represent the simulation outputs with lower and higher reproductive ratios of the wild type with *R*_e_ = 1.1 and 1.3, respectively. (**B**) Overall and age-specific relative reduction of attack rates with vaccination, as compared to the outbreak scenario without further vaccination over 150 days.

Without the emergence of a SARS-CoV-2 variant, we found that the vaccination program against COVID-19 in South Korea would avert 45% of symptomatic infections with a baseline scenario (Figure 2). The highest relative reduction (55–56%) was observed among individuals aged 40–59 years. We found that with an increased transmission potential, i.e., *R*_e_ = 1.3, the vaccination program would reduce the attack rate from 9.2% to 4.9%, averting 47% of symptomatic infections.

**Figure 3:**
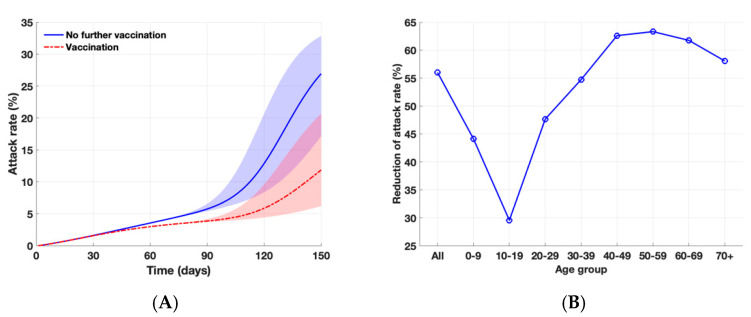
Impact of vaccination program against COVID-19 during the fourth wave in the presence of SARS-CoV-2 variant. The shaded areas represent the simulation outputs with lower and higher reproductive ratios of the Delta variant with υ=1.8 and υ=2.2, respectively (**A**) Projected cumulative attack rates over 150 days with and without further vaccination. (**B**) Overall and age-specific relative reduction of attack rates with vaccination, as compared to the outbreak scenario without further vaccination over 150 days.

We found that, in the absence of vaccination and the emergence of the Delta variant, the attack rate would be 26.9% over 150 days (Figure 3), compared to 6.9% in the absence of a SARS-CoV-2 variant. Furthermore, the variant would become a dominant strain within 65 days after introduction. With the emergence of a SARS-CoV-2 variant in at the end of July, assuming a baseline transmission potential of the Delta variant, the vaccination program in South Korea would reduce cumulative symptomatic infections by 56% over the same period. As vaccine coverage level increases over time, the larger benefits of a vaccination program in averting symptomatic infections would be achieved especially in the presence a SARS-CoV-2 variant; however, herd immunity is unlikely to be achieved.

### 3.2. Hospitalization and Deaths

**Figure 4:**
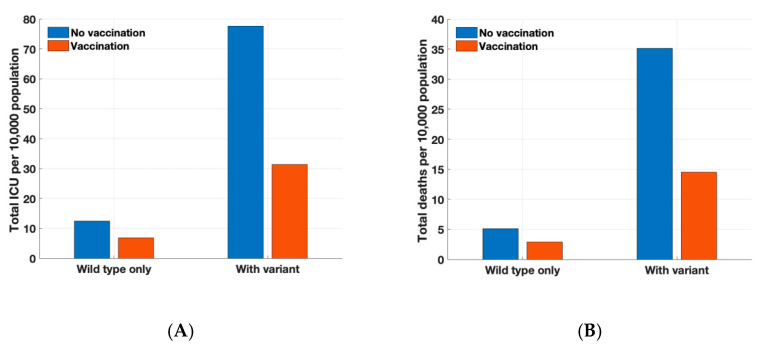
Projected total number of (**A**) ICU hospitalizations and (**B**) deaths per 10,000 persons over 150 days.

In the absence of vaccination, total intensive care unit (ICU) hospitalizations and deaths were projected to be 12.4 and 5.1 per 10,000 persons, respectively, on day 150 without the emergence of variant strains. Vaccination with reduced efficacy in elderly individuals still markedly reduced hospitalizations and deaths (Figure 4). Vaccination reduced infection outcomes, such as ICU hospitalizations and deaths, decreasing it by 45% and 43%, respectively. When we consider the situation after emergence of the Delta variant, further reduction in hospitalizations and deaths can be expected. In this scenario, total ICU hospitalizations and deaths were projected to be 77.6 and 35.1 per 10,000 persons, respectively, on day 150 without vaccines. Vaccination would reduce total ICU hospitalizations and deaths to 31.3 and 14.5 per 10,000 persons, respectively.

## 4. Discussion

Vaccination is expected to have a substantial impact in mitigating COVID-19 outbreaks [40]. However, owing to challenges such as vaccine supply and rollout, coupled with the potential emergence of highly transmissible SARS-CoV-2 variants, it is debatable whether the current vaccination strategy would generate herd immunity. It has been also suggested that available vaccines should be used for a single-dose COVID-19 vaccination strategy rather than a two-dose regimen, to double vaccine coverage. A few countries, including the UK and Canada, have approved guidelines to defer the second dose by up to 12 and 16 weeks, respectively [16]. The South Korean government announced plans to defer the second dose by up to 12 weeks, and aimed to vaccinate 36 million people with their first doses by September 2021. In this study, we evaluated the impact of COVID-19 vaccination program in South Korea considering the potential emergence of SARS-CoV-2 variants and its impact on the vaccination program.

Our results indicate that a SARS-CoV-2 variant with higher transmissibility compared to the currently circulating strain would become dominant within two months of being introduced. Our findings are consistent with prior studies that indicated that the variant alpha would become the predominant variant in the US, 2–3 months after introduction [3,41]. Even in the presence of SARS-CoV-2 variants, a vaccination program was shown to have potential in significantly reducing the disease burden associated with COVID-19. We project that fully vaccinating 33 million people against COVID-19 would avert at least 45% of symptomatic infections, 45% of hospitalizations, and 43% of deaths. Nevertheless, we found that herd immunity is unlikely to be achieved by the end of November 2021 with the emergence of the Delta variant, in contrast to the country’s vaccination blueprint with a goal to achieve herd immunity by November 2021.

Our study has some limitations. First, we did not explicitly model high-risk individuals including healthcare workers and comorbid individuals. Inclusion of those individuals may improve the accuracy of model predictions. Second, our parameter values for transmissibility of SARS-CoV-2 variants and the vaccine efficacy against it are based on early reports of the Delta variant in the UK; however, these estimates may vary across different populations or settings.

## 5. Conclusions

Our study unequivocally suggests a substantial impact of vaccination program on mitigating the severity of COVID-19 infection at the population level in South Korea. However, this strategy is unlikely to help achieve herd immunity with the potential emergence of more contagious SARS-CoV-2 variants, as the current pace of vaccine rollout is insufficient to prevent the exacerbation of cases, hospitalizations, and deaths expected.

## Data Availability

The daily number of confirmed cases and deaths associated with COVID-19 in South Korea was obtained from publicly available sources, available at https://www.kdca.go.kr (accessed on 5 July 2021).
